# Silicon photomultiplier‐based scintillation detectors for photon‐counting CT: A feasibility study

**DOI:** 10.1002/mp.14886

**Published:** 2021-06-25

**Authors:** Stefan J. van der Sar, Stefan E. Brunner, Dennis R. Schaart

**Affiliations:** ^1^ Department of Radiation Science and Technology Delft University of Technology Delft The Netherlands; ^2^ Broadcom Inc. Regensburg Germany; ^3^ Holland Proton Therapy Center Delft The Netherlands

**Keywords:** energy resolution, photon‐counting computed tomography, pulse shape, scintillator, silicon photomultiplier

## Abstract

**Purpose:**

The implementation of photon‐counting detectors is widely expected to be the next breakthrough in X‐ray computed tomography (CT) instrumentation. A small number of prototype scanners equipped with direct‐conversion detectors based on room‐temperature semiconductors, such as CdTe and CdZnTe (CZT), are currently installed at medical centers. Here, we investigate the feasibility of using silicon photomultiplier (SiPM)‐based scintillation detectors in photon‐counting computed tomography (PCCT) scanners, as a potential alternative to CdTe and CZT detectors.

**Methods:**

We introduce a model that allows us to compute the expected energy resolution as well as the expected pulse shape and associated rate capability of SiPM‐based PCCT detectors. The model takes into account SiPM saturation and optical crosstalk, because these phenomena may substantially affect the performance of SiPM‐based PCCT detectors with sub‐mm pixels. We present model validation experiments using a single‐pixel detector consisting of a 0.9 × 0.9 × 1.0 mm^3^ LuAP:Ce scintillation crystal coupled to a 1 × 1 mm^2^ SiPM. We subsequently use the validated model to compute the expected performance of the fast scintillators LYSO:Ce, LuAP:Ce, and LaBr_3_:Ce, coupled to currently available SiPMs, as well as to a more advanced SiPM prototype with improved dynamic range, for sub‐mm pixel sizes.

**Results:**

The model was found to be in good agreement with the validation experiments, both with respect to energy resolution and pulse shape. It shows how saturation progressively degrades the energy resolution of detectors equipped with currently available SiPMs as the pixel size decreases. Moreover, the expected pulse duration is relatively long (~200 ns) with these SiPMs. However, when LuAP:Ce and LaBr_3_:Ce are coupled to the more advanced SiPM prototype, the pulse duration improves to less than 60 ns, which is in the same order of magnitude as pulses from CdTe and CZT detectors. It follows that sufficient rate capability can be achieved with pixel sizes of 400 μm or smaller. Moreover, LaBr_3_:Ce detectors can provide an energy resolution of 11.5%‐13.5% at 60 keV, comparable to CdTe and CZT detectors.

**Conclusions:**

This work provides first evidence that it may be feasible to develop SiPM‐based scintillation detectors for PCCT that can compete with CdTe and CZT detectors in terms of energy resolution and rate capability.

## 1 INTRODUCTION

The implementation of photon‐counting X‐ray detectors is widely expected to be the next technological breakthrough in CT scanner development. Several vision and review papers were published in the last 10 years.^1^, ^2^, ^3^, ^4^ These highlight the potential benefits of replacing conventional energy‐integrating detectors by photon‐counting detectors. For example, the image quality (contrast‐to‐noise ratio) can be improved and/or the radiation dose and contrast agent load can be reduced. Moreover, truly simultaneous acquisition of dual‐energy data with good spectral separation becomes possible. Even multi‐energy data can be acquired, which opens up opportunities to perform K‐edge imaging, for example. Photon‐counting detectors enable such benefits by measuring the number of X‐ray photons and assigning each X‐ray photon to one of a finite number of energy bins. To do so accurately, detectors must be able to handle an incident X‐ray photon fluence rate in the order of 10^2^ Mcps/mm^2^ and have sufficient energy resolution. Therefore, direct‐conversion detectors with sub‐mm pixels based on room‐temperature semiconductors with a high density and a high atomic number, such as CdTe and CdZnTe (CZT), are generally considered promising for this application. Specific drawbacks of these detectors are the occurrence of charge sharing and charge trapping. The latter can lead to unstable and unreliable detector operation.^1^, ^4^ High purity materials with a very low defect concentration are required to reduce this effect. This may negatively affect the cost‐effectiveness of production. Nevertheless, a small number of prototype scanners equipped with CdTe or CZT detectors have been installed at medical centers to investigate the benefits of photon‐counting computed tomography (PCCT) in clinical practice. The first results were published in 2016.^5^, ^6^ An overview of studies conducted since then can be found in a recent review paper.^4^

Scintillation detectors could be an alternative to the aforementioned direct‐conversion detectors. Although the latter type of detector is commonly considered most suitable for applications that require short pulse duration and good energy resolution,^7^ scintillation detectors are successfully employed in most commercial radiological and nuclear medicine imaging systems. In this contribution, we aim to show that fast scintillators with state‐of‐the‐art energy resolution, in combination with recent developments in silicon photomultiplier (SiPM) technology, may enable the application of scintillation detectors in PCCT scanners. We do so by describing the basic principles of SiPM‐based scintillation detectors in Section 2. In the same section, we introduce a model that allows us to compute two fundamental properties of such detectors for PCCT, viz. the rate capability and the (low‐rate) energy resolution. The model considers the raw detector pulses. In other words, no particular way of pulse processing and counting is assumed. In Sections 3 and 4, we experimentally validate the model and use it to compute the expected performance of SiPM‐based scintillation detectors with sub‐mm pixels for PCCT. We discuss the results and draw conclusions in Sections 5 and 6.

## 2 THEORY

**Fig. 1:**
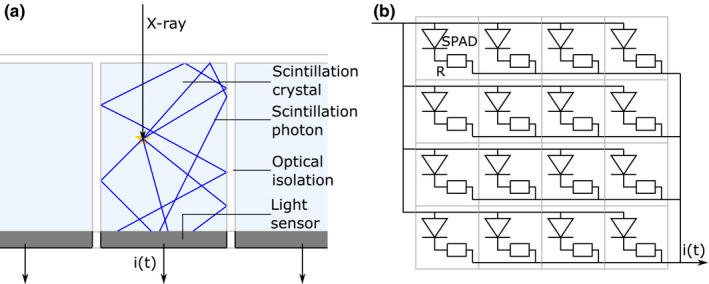
(a) Schematic cross‐section of a pixelated scintillation detector for PCCT. Each scintillation crystal is one‐to‐one coupled to a light sensor, such as a silicon photomultiplier (SiPM), which converts the pulse of optical photons generated by the interaction of an X‐ray photon in the scintillator into a current pulse *i(t)*. The paths of five scintillation photons are represented here by the blue lines; in reality the number of photons is much larger. (b) Schematic top view of an SiPM, a light sensor that consists of a two‐dimensional array of single‐photon avalanche diodes (SPADs). The SPADs of a single SiPM are connected in parallel and each SPAD is equipped with a quenching resistor (R). Only a 4 × 4 array of SPADs is shown here; practical SiPMs consist of 10^2^‐10^5^ SPADs. Each detector pixel consists of a scintillation crystal coupled to its own SiPM, that is, to its own two‐dimensional array of SPADs, which generates the current pulse *i(t)*.

Scintillation detectors are also known as indirect‐conversion detectors, because a scintillator first converts an X‐ray photon into a tiny light pulse, which in turn is converted into a current pulse by a light sensor. Obviously, a pixelated detector is needed for CT imaging. As shown schematically in Fig. 1(a), this can be achieved by optically coupling an array of scintillation crystals one‐to‐one to an array of light sensors. Some form of optical isolation between the individual crystals is useful to guide the optical photons toward the sensor and to prevent light sharing between pixels. It is noted that conventional reflectors applied to sub‐mm pixels can result in a relatively large dead area and a loss of dose efficiency (see Section 5). On the other hand, optical photons rather than charge carriers are transported in a scintillation detector, so typical issues associated with direct‐conversion detectors, such as charge sharing and charge trapping, do not play a role. The escape of secondary X‐rays from sub‐mm pixels may occur in both types of detector (also see Section 5).

Various types of light sensor are available.^8^ Photodiodes are used in the energy‐integrating scintillation detectors of conventional CT scanners. Since photodiodes do not provide internal amplification (gain), the signal‐to‐noise ratio (SNR) of the detector output signal in response to a single X‐ray photon tends to be poor, which makes them unsuitable for photon‐counting detectors. Photomultiplier tubes (PMTs) provide a high internal gain (in the order of 10^6^–10^8^), but do not offer sub‐mm pixelated readout as required for photon‐counting CT. The silicon photomultiplier (SiPM) is a more recent type of light sensor, which has successfully replaced PMTs in commercial positron emission tomography (PET) scanners.^9^ SiPMs combine high internal gain (typically 10^6^ at a bias voltage of only a few tens of volts) with the possibility of pixel miniaturization. We therefore choose the SiPM as the light sensor for photon‐counting CT.

**Fig. 2:**
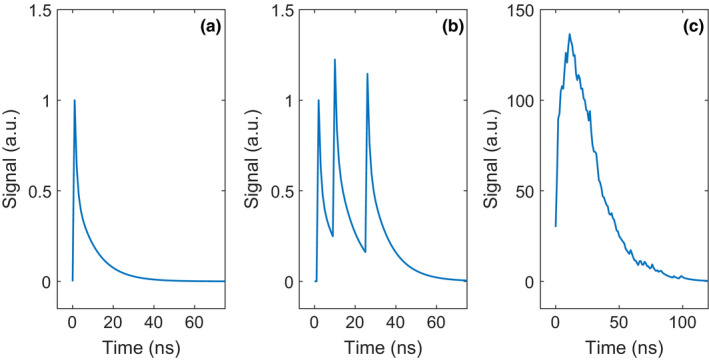
(a) A typical SiPM output pulse in response to a single trigger (single‐SPAD response, SSR). (b) A typical SiPM output pulse in response to three triggers in three different SPADs at three different moments in time. (c) A typical SiPM output pulse in response to an X‐ray interaction in a scintillator, that is, in response to many (e.g., 750) triggers occurring at different moments in time distributed according to equation (2).

As shown in Fig. 1(b), an SiPM consists of a two‐dimensional array of single‐photon avalanche diodes (SPADs).^10^ These are small photodiodes (pitch ≤100 μm) that are reverse biased a few volts above their breakdown voltage. Consequently, the electron–hole pair created by the absorption of an optical photon in one of the SPADs can trigger an avalanche multiplication process. When that happens, the SPAD is said to fire, or discharge. The rapidly increasing current through the quenching resistor causes the voltage across the diode to drop, resulting in the quenching of the avalanche. Thus, SPADs are photodiodes operated in Geiger mode and the total amount of charge released in response to a single trigger is nearly constant. Fig. 2(a) shows the resulting pulse from the SiPM (the single‐SPAD response, or SSR), which can be described as a fast spike (few ns width) followed by an exponential decay characterized by the recharge time constant *τ*_r_.^11^ Typical values of *τ*_r_ are in the order of 10^1^ ns. All of the SPADs on an SiPM are connected in parallel (see Fig. 1(b)). Thus, if multiple optical photons trigger avalanches in multiple SPADs, all of these SPADs fire and the resulting SiPM output pulse equals the superposition of the pulses from the individual SPADs. An example of the SiPM output pulse resulting from three fired SPADs is shown in Fig. 2(b).

### 2.A Pulse formation in SiPM‐based scintillation detectors

Following an X‐ray interaction in a scintillator, many optical photons will be detected by the SiPM. The expected number of scintillation photon‐induced triggers ${\bar{n}}_{\text{tr},\text{sc}}$ is given by:

(1) $${\bar{n}}_{\text{tr},\text{sc}}\;=\;E\cdot Y\cdot f\cdot \eta_{\text{lc}}\cdot \eta_{\text{pd}}$$

Here, *E* is the deposited X‐ray energy in the scintillator [keV] and *Y* is the light yield of the scintillator [photons/keV]. *Y* is often a function of *E*. This phenomenon is called light yield nonproportionality. Values of *Y* are commonly measured at 662 keV. Nonproportionality factors *f*, describing the ratio between the value of *Y* at the energy of interest and at 662 keV, must then be known to calculate the absolute photon yield at other energies. The light collection efficiency *η*_lc_ refers to the fraction of scintillation photons that reaches the SiPM. Only a fraction of these photons triggers avalanches. This is quantified by the photodetection efficiency *η*_pd_. For detected X‐rays in the diagnostic energy range, ${\bar{n}}_{\text{tr},\text{sc}}$ is typically in the order of 10^2^–10^3^.

Scintillation photons are not emitted instantaneously upon the interaction of an X‐ray photon. In first‐order approximation, the probability *P*(Δ*t*) that a scintillation photon is emitted a period of time Δ*t* after the X‐ray interaction can be assumed to follow an exponential distribution, characterized by the scintillation decay time constant *τ*_d_:

(2) $$P\left(\Delta t\right)\;=\;\frac{1}{\tau_{d}}\exp\left(‐\frac{\Delta t}{\tau_{d}}\right)$$

Fast scintillators may have a decay time constant in the order of ns, whereas *τ*_d_ may be in the order of μs or more for a slow scintillator, such as GOS, which is used in energy‐integrating CT detectors (*τ*_d_ ~ 2.5 µs). The triggering of the SPADs can be assumed to follow the same probability distribution (equation (2)), because the transfer time spread of optical photons in sub‐mm scintillation crystals is typically much smaller than *τ*_d_. Fig. 2(c) shows an example of the resulting SiPM output pulse in response to an X‐ray interaction in the scintillator. Due to the high internal gain of SiPMs, the use of charge‐sensitive amplifiers and carefully optimized pulse‐shaping circuitry, as required for direct‐conversion detectors, is no longer mandatory. Good results can be obtained, for example, by feeding the SiPM current pulses directly into a trans‐impedance amplifier and performing pulse height analysis on the resulting voltage pulses.

### 2.B Noise sources in SiPMs

Thermal energy can release charge carriers that trigger discharges in SPADs. These are called dark triggers. A dark trigger gives rise to the same signal as shown in Fig. 2(a), that is, the single‐SPAD response. Such a pulse is much weaker than a typical X‐ray induced pulse (Fig. 2(c)), so it typically does not cross the counting threshold of a photon‐counting X‐ray detector. If a dark trigger occurs during an X‐ray induced pulse, the effect on that pulse is very small. Moreover, the typical rate of dark triggers in modern SiPMs is in the order of 10^4^‐10^5^ per second per mm^2^, which is much lower than the typical rate of X‐ray photons, so most X‐ray induced pulses will not at all be affected by dark triggers.

Another source of noise is afterpulsing. This concerns delayed avalanches triggered by trapped charge carriers escaping their traps sometime after an optical photon‐induced discharge. Afterpulses rarely occur in modern SiPMs and their effect on an X‐ray photon‐counting detector is similar to the effect of dark triggers, so they are neglected in this research.

When a SPAD fires, some of the charge carriers involved in the avalanche multiplication process produce infrared photons. These can nearly instantaneously trigger avalanches in nearby SPADs on the same SiPM. Each of these secondary triggers in turn can cause tertiary triggers, and so on. This phenomenon is called optical crosstalk. The probability that a single trigger causes a total of *n*_tr,oc_ triggers (including itself) is given by the Borel distribution with parameter *λ*:^12^

(3) $$P\left(n_{\text{tr},\text{oc}}\right)=\frac{{\left(\lambda \cdot n_{\text{tr},\text{oc}}\right)}^{n_{\text{tr},\text{oc}}‐1}\cdot \exp\left(‐\lambda \cdot n_{\text{tr},\text{oc}}\right)}{n_{\text{tr},\text{oc}}!}$$

**Fig. 3:**
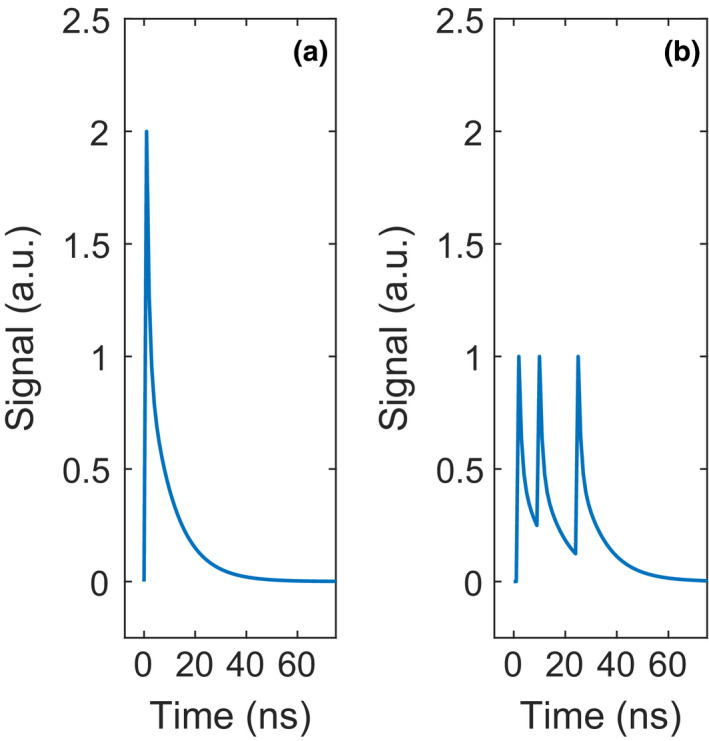
(a) A typical SiPM output pulse in response to a single scintillation photon‐induced trigger (or dark trigger) causing one crosstalk photon‐induced trigger. (b) A typical SiPM output pulse in response to three triggers in the same SPAD shortly after one another, giving rise to partial SPAD pulses.

The physical meaning of *λ* is the average number of directly‐succeeding triggers caused by a single preceding trigger. Under normal operation conditions, 0 < *λ <* 1, but more typically, 0 < *λ <* 0.5. Fig. 3(a) shows the typical SiPM output pulse for the case of a single scintillation photon‐induced trigger (or dark trigger) causing one crosstalk photon‐induced trigger. The pulse amplitude and integral are twice those of the single‐SPAD response (Fig. 2(a)).

If the number of scintillation photon‐induced triggers is assumed to be Poisson distributed with mean ${\bar{n}}_{\text{tr},\text{sc}}$ given by Equation (1), the probability of having *n*_tr,tot_ triggers in total (scintillation photon‐ plus crosstalk photon‐induced triggers) will follow a generalized Poisson distribution:^12^

(4) $$P\left(n_{\text{tr},\text{tot}}\right)=\frac{{\bar{n}}_{\text{tr},\text{sc}}\cdot {\left({\bar{n}}_{\text{tr},\text{sc}}+\lambda \cdot n_{\text{tr},\text{tot}}\right)}^{n_{\text{tr},\text{tot}}‐1}\cdot \exp\left(‐{\bar{n}}_{\text{tr},\text{sc}}‐\lambda \cdot n_{\text{tr},\text{tot}}\right)}{n_{\text{tr},\text{tot}}!}$$

This distribution has the following mean value:^12^

(5) $${\bar{n}}_{\text{tr,tot}}=\frac{{\bar{n}}_{\text{tr,sc}}}{1‐\lambda }$$

and variance:^12^

(6) $$\text{var}\left(n_{\text{tr,tot}}\right)=\frac{{\bar{n}}_{\text{tr,sc}}}{{\left(1‐\lambda \right)}^{3}}$$

Equations (5) and (6) not only show that optical crosstalk increases the total number of triggers, but also that it comes at the cost of a more strongly increasing variance in the total number of triggers.

### 2.C Nonproportional response of SiPMs

Sub‐mm pixels, required to handle the high incident fluence rate of X‐ray photons in photon‐counting CT, can accommodate a limited number of SPADs. This makes it more likely that two or more optical photons fire the same SPAD, which can lead to the phenomenon of partial SPAD pulses as shown in Fig. 3(b). A partial pulse occurs when an optical photon triggers a new avalanche in a SPAD before that SPAD has fully recharged after the previous discharge.^13^ A quantity called the equivalent number of fired SPADs, *n*_f,eq_, can be obtained by dividing the integral of an SiPM output pulse by the integral of the single‐SPAD response (Fig. 2(a)). For three triggers in three different SPADs (Fig. 2(b)), *n*_f,eq_ equals 3, whereas it is less than 3 for three triggers occurring shortly after one another in the same SPAD (Fig. 3(b)). Due to the occurrence of partial pulses, the relationship between *n*_f,eq_ and the number of scintillation photon‐induced triggers *n*_tr,sc_ becomes supra‐proportional. As the number of partial pulses in an SiPM output pulse increases, the deviation from proportional behavior increases and the SiPM is said to saturate.

Van Dam et al.^13^ developed an analytical expression for the nonproportional relationship between the expected value ${\bar{n}}_{\text{f,eq}}$ of the equivalent number of fired SPADs and the expected value ${\bar{n}}_{\text{tr,sc}}$ of the Poisson‐distributed number of scintillation photon‐induced triggers given by Equation (1). They took into account afterpulsing and optical crosstalk (both of which tend to increase ${\bar{n}}_{\text{f,eq}}$), as well as saturation (which tends to decrease ${\bar{n}}_{\text{f,eq}}$) and showed that their model was in good agreement with experimental data up to very high saturation levels.^13^ Here, we use a slightly adapted version of Van Dam’s model that is briefly explained in the following.

1. According to Equation (5), ${\bar{n}}_{\text{tr,sc}}$ scintillation photon‐induced triggers on average give rise to ${\bar{n}}_{\text{tr,tot}}$ triggers in total. Note that our use of Equation (5) to calculate ${\bar{n}}_{\text{tr,tot}}$ may be more accurate than the approximation originally used by Van Dam et al.,^13^ in particular when the crosstalk parameter *λ* is large.
2. Assuming a uniform distribution of ${\bar{n}}_{\text{tr,tot}}$ triggers over the number of SPADs *N*_SPAD_, the probability *P*(*i*) that a single SPAD is triggered *i* times is given by a binomial distribution with ${\bar{n}}_{\text{tr,tot}}$ trials and a success probability of 1/*N*_SPAD_. This can be approximated by a Poisson distribution with a mean value of ${\bar{n}}_{\text{tr,tot}}$/*N*_SPAD_.
3. If afterpulsing is neglected, the expression in Van Dam et al.^13^ for the expected value ${\bar{n}}_{\text{f,eq,1}}$ (*i*) of the equivalent number of fired SPADs for a single SPAD that is triggered *i* times reduces to:

(7) $${\bar{n}}_{\text{f,eq,1}}\left(i\right)\;=\;{\bar{n}}_{\text{f,eq,1}}\left(i‐1\right)+1‐\frac{\left(i‐1\right)\tau_{r}}{\left(i‐1\right)\tau_{r}+\tau_{d}}\;\text{with}\;{\bar{n}}_{\text{f,eq,1}}\left(1\right)=1$$

It can be appreciated that ${\bar{n}}_{\text{f,eq,1}}\left(i\right)$ = *i* for an infinitely short SPAD response (i.e., *τ*_r_→0). In other words, no saturation occurs in that case. It is noted that the fast spike in the single‐SPAD response (see Fig. 2(a)) was neglected to arrive at Equation (7), as this allows the single‐SPAD response to be modeled by a single‐exponential decay function with time constant *τ*_r_.

- The expected value ${\bar{n}}_{\text{f,eq,1}}$ of the equivalent number of fired SPADs for a single SPAD is now given by the weighted average of the ${\bar{n}}_{\text{f,eq,1}}\left(i\right)$ with weights *P*(*i*):

(8) $${\bar{n}}_{\text{f,eq,1}}=\sum_{i=1}^{\infty }P\left(i\right)\cdot {\bar{n}}_{\text{f,eq,1}}\left(i\right)$$

- The expected value ${\bar{n}}_{\text{f,eq}}$ of the equivalent number of fired SPADs for all SPADs on the SiPM can then be obtained by multiplying with *N*_SPAD_:

(9) $${\bar{n}}_{\text{f,eq}}=N_{\text{SPAD}}\cdot {\bar{n}}_{\text{f,eq,1}}$$

- Equation (9) will increasingly overestimate ${\bar{n}}_{\text{f,eq}}$ as the saturation level increases, because fewer optical crosstalk photons are emitted in case of a partial discharge and fewer crosstalk photon‐induced triggers will occur. Van Dam et al.^13^ showed that this can be dealt with by iteratively solving the following equation for the corrected expected value ${\bar{n}}_{\text{tr},\text{tot},\text{corr}}$ of the total number of triggers:

(10) $${\bar{n}}_{\text{tr},\text{tot},\text{corr}}=\frac{{\bar{n}}_{\text{tr},\text{sc}}}{1‐\lambda \cdot \frac{{\bar{n}}_{\text{f},\text{eq}}}{{\bar{n}}_{\text{tr},\text{tot},\text{corr}}}}$$

where ${\bar{n}}_{\text{tr,tot}}$, given by Equation (5), is the initial guess for ${\bar{n}}_{\text{tr},\text{tot},\text{corr}}$ in the iterative process. The value to which ${\bar{n}}_{\text{tr},\text{tot},\text{corr}}$ converges is then used to recalculate ${\bar{n}}_{f,\text{eq}}$.

### 2.D Pulse shape model

In this paper, we introduce an extension of the above‐described model by Van Dam et al.^13^ The extension not only enables the calculation of the mean of the equivalent number of fired SPADs (${\bar{n}}_{\text{f,eq}}$), but also the distribution of that number, which is needed to compute the expected energy resolution of SiPM‐based scintillation detectors. We do so by simulating many (e.g., 10^4^) output pulses from a given combination of scintillator and SiPM, in response to a given energy deposition, according to the principles and assumptions of van Dam’s model as described in Section 2.C. This will also enable us to evaluate the expected pulse duration and associated rate capability. The simulation steps are as follows:

1. Given an X‐ray energy deposition *E*, a set of scintillator properties (*τ*_d_, *Y*, *f)*, a value for the light collection efficiency *η*_lc_, and a set of SiPM properties (*η*_pd_, *τ*_r_, *λ*, *N*_SPAD_), calculate ${\bar{n}}_{\text{tr,sc}}$ and ${\bar{n}}_{\text{tr,tot}}$ using Equation (1) and (5), respectively. Here, it is assumed that *N*_SPAD_ equals the pixel area divided by the SPAD pitch squared.
2. Use van Dam’s model as described in Section 2.C to determine ${\bar{n}}_{\text{f,eq}}$ and ${\bar{n}}_{\text{tr,tot,corr}}$, such that a corrected value for *λ* can be calculated:

(11) $$\lambda_{\text{corr}}=\lambda \cdot \frac{{\bar{n}}_{\text{f,eq}}}{{\bar{n}}_{\text{tr,tot,corr}}}$$

**Fig. 4:**
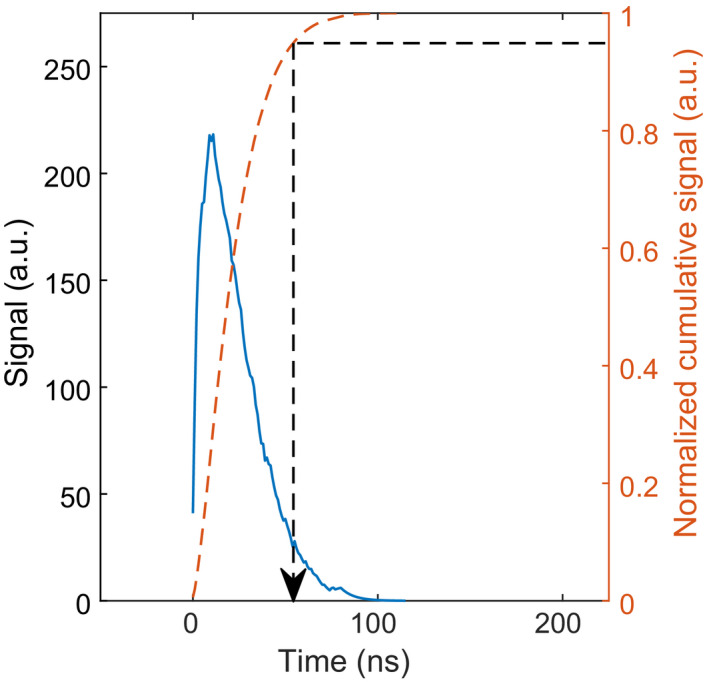
A typical output pulse from an SiPM‐based scintillation detector in response to an X‐ray photon interaction in a scintillator, calculated with the model presented in this article (solid blue curve). The dashed orange curve shows the normalized cumulative signal. The pulse duration is defined as the moment in time at which the normalized cumulative signal reaches a value of 0.95 (black dashed arrow). The simulated detector consists of a LaBr_3_:Ce scintillator (see Table III) coupled to an 0.4 × 0.4 mm^2^ SiPM with a SPAD pitch of 15 µm (see Table IV), exposed to 60 keV photons.

- Sample a number of triggers from a generalized Poisson distribution with parameters ${\bar{n}}_{\text{tr,sc}}$ and *λ*_corr_ (Equation (4)). This can be done using Sterling’s approximation with three terms and the inverse transform sampling method, for example.
- Assign each trigger to a SPAD by sampling from a uniform distribution of integers on the interval [1, *N*_SPAD_]. As a result, the number of triggers on each SPAD is known.
- For each of the SPADs, sample the timestamps of its triggers from the scintillator emission function, that is, Equation (2), and use these to construct the total SPAD signal. Fig. 3(b) shows an example of such a signal. However, note that we model the single‐SPAD response (cf. Fig. 2(a)) as a single‐exponential decay function characterized by the recharge time constant *τ*_r_, so as to be consistent with Van Dam et al.^13^ (see also Equation (7) and accompanying text).
- Add up all SPAD signals to obtain the detector output pulse. An example is shown in Fig. 4.
- Repeat steps 3, 4, 5, and 6 until the desired number of output pulses has been simulated.

### 2.E Model output I: Energy resolution

For each simulated output pulse, we obtain the equivalent number of fired SPADs $n_{\text{f,eq}}$ by dividing the integral of the output pulse by the integral of the single‐SPAD response. We subsequently make a histogram of $n_{\text{f,eq}}$ of all simulated pulses and fit it using a Gaussian function. In this way, we determine the mean (${\bar{n}}_{\text{f,eq}}$) and the full width at half maximum (FWHM, $\Delta n_{\text{f,eq}}$). We then calculate the observed energy resolution *R*_obs_ as:

(12) $$R_{\text{obs}}=\frac{\Delta n_{\text{f,eq}}}{{\bar{n}}_{\text{f,eq}}}\cdot 100\%$$

**Fig. 5:**
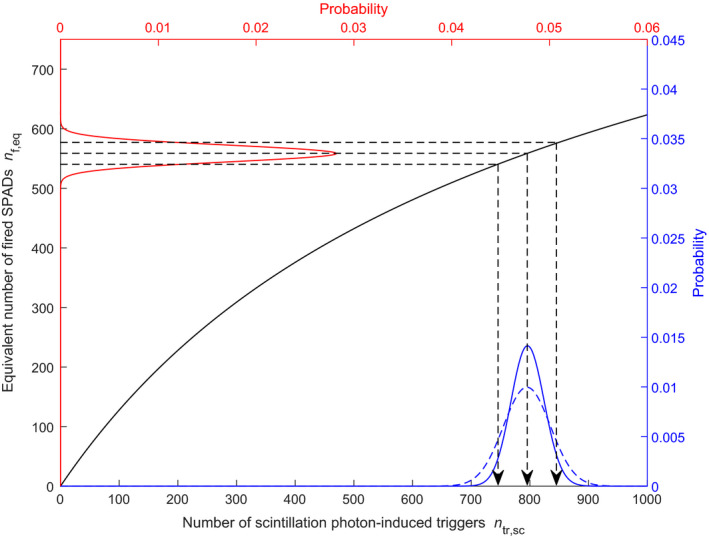
Given a Poisson‐distributed number of scintillation photon‐induced triggers *n*_tr,sc_ (solid blue curve on the horizontal axis) with mean ${\bar{n}}_{\text{tr},\text{sc}}$ given by equation (1), we first compute the expected distribution of the equivalent number of fired SPADs *n*_f,eq_ as described in Sections 2.D and 2.E (solid red curve on the vertical axis). The latter distribution is subsequently “backprojected” to the domain of *n*_tr,sc_ (dashed black lines), using van Dam’s model of the nonproportional SiPM response described in Section 2.C (solid black curve). The corrected energy resolution *R*_corr_ obtained from the resulting distribution (dashed blue curve on the horizontal axis) is clearly worse than expected from Poisson statistics, due to the nonproportional SiPM response.

Note that *R*_obs_ does not represent the true energy discrimination capability of the detector, because *n*_f,eq_ is not proportional to the number of scintillation photon‐induced triggers *n*_tr,sc_ (see Section 2.C) and, therefore, not proportional to the X‐ray energy (see Equation (1)). Consequently, the simulated data must be “backprojected” to the domain of *n*_tr,sc_, as illustrated in Fig. 5. The distribution on the vertical axis is an example of a simulation result in the domain of *n*_f,eq_. The values of *n*_f,eq_ corresponding to the mean and the FWHM of this distribution are backprojected to the domain of *n*_tr,sc_ (dashed black lines). The backprojection is based on a look‐up table (visualized by the solid black curve) calculated using the analytical expression of Van Dam’s model described in Section 2.C. This yields values for the FWHM and the mean in the domain of *n*_tr,sc_. The ratio of these two values is considered the corrected energy resolution *R*_corr_, in analogy with Equation (12). A comparison of the distributions on the horizontal axis shows that *R*_corr_ is worse than expected from a Poisson distribution with mean ${\bar{n}}_{\text{tr,sc}}$ (as given by Equation (1)) due to the nonproportional SiPM response (optical crosstalk, saturation). In addition, *R*_corr_ degrades more and more as the saturation level increases. *R*_obs_ shows the opposite behavior, which gives a misleading impression of the effect of saturation.

It must be noted that the true energy resolution *R* of a scintillation detector consists of several components:^14^

(13) $$R^{2}=R_{\text{stat}}^{2}+R_{\text{intr}}^{2}=R_{\text{stat}}^{2}+R_{\text{np}}^{2}+R_{\text{inh}}^{2}+R_{\text{tr}}^{2}$$

Here, *R*_stat_ refers to the statistics of scintillation photon detection and this is the component that we compute with our model, that is, *R*_corr_ is the expected *R*_stat_. We obtain the expected *R* by Pythagorean addition of the intrinsic component *R*_intr_, which combines all other contributions to the energy resolution. The most important of these contributions, *R*_np_, is the excess variance caused by the nonproportionality of the scintillator’s light yield. The second contribution, *R*_inh_, is the excess variance due to inhomogeneity of the scintillator, for example, spatial variation of the light yield. The last contribution, *R*_tr_, is the excess variance resulting from the light transport. Since *R*_intr_ cannot be modeled from first principles, we rely on measured values reported in literature. These values are mainly determined by *R*_np_, because *R*_inh_ and *R*_tr_ should be negligible for well‐constructed detectors based on commercially grown scintillation crystals, particularly in the case of small pixels.

### 2.F Model output II: Pulse duration and rate capability

The expected pulse duration and associated rate capability of a given combination of scintillator and SiPM can be derived from the set of output pulses already simulated in Section 2.D. In this study, we define the duration of a detector output pulse as the moment in time at which the integral of the pulse reaches 95% of the total area under the pulse (see Fig. 4). With this definition, we calculate the mean *t*_95_ of all pulse durations in the set. Then, we calculate the incident rate *r*_50_,_pix_ at which an X‐ray photon has 50% chance of arriving within *t*_95_ from the previous X‐ray photon on the same pixel, in which case the resulting pulse is considered to be affected by pulse pile‐up. Note that at the rate *r*_50_,_pix_ there is also 50% chance for a given event to occur more than *t*_95_ later than the previous one, in which case the pulse is considered not to be affected by pile‐up. Given the exponential distribution of the inter‐arrival times of the X‐ray photons, *r*_50,pix_ is given by:

(14) $$r_{50,\text{pix}}=\frac{\ln(2)}{t_{95}}$$

Finally, we translate *r*_50_,_pix_ into a rate capability per mm^2^, denoted by *r*_50_, using the pixel size *d* in mm:

(15) $$r_{50}=r_{\text{50,pix}}\cdot {\left(\frac{1}{d}\right)}^{2}$$

## 3 MATERIALS AND METHODS

We first describe the experiments conducted to validate the model introduced in Section 2. We then describe the simulations performed to investigate the feasibility of developing SiPM‐based scintillation detectors for use in photon‐counting CT scanners.

### 3.A Model validation: Experimental set‐up

A noncommercial 1 × 1 mm^2^ SiPM from Broadcom based on the manufacturer’s NUV‐HD technology was used in the validation experiments. It was optically coupled to a fast 0.9 × 0.9 × 1.0 mm^3^ LuAlO_3_:Ce (LuAP:Ce) scintillation crystal (Hilger Crystals, *τ*_d_ = 17 ns) using Norland Optical Adhesive 63. Note that due to the high effective atomic number and density of LuAP:Ce (*Z*_Lu_ = 71 and *ρ*_LuAP_ = 8.3 g cm^−3^), a crystal thickness of only 1.0 mm provides X‐ray detection efficiencies of about 96%, 91%, and 58% at 50, 100, and 150 keV, respectively. The scintillation crystal was covered in reflective polytetrafluoroethylene (PTFE) powder in order to increase the light collection efficiency. Two radionuclides were selected for the measurements: Am‐241, with a single emission line at 59.5 keV, and Cs‐137, with a single emission line at 662 keV. Although the latter energy is outside the diagnostic energy range, it helped to create a relatively high saturation level on the 1×1 mm^2^ SiPM, such that we could also test our model under these potentially relevant conditions for sub‐mm pixels. The current pulses from the detector were converted into voltage pulses, without substantial changes to the pulse shape, using a trans‐impedance amplifier on Broadcom’s AFBR‐S4E001 preamplifier board. In the next step, the pulses were digitized by a Teledyne LeCroy HDO9404 digital oscilloscope operating at a bandwidth of 200 MHz and a sampling rate of 1 GS/s. In this way, further analysis of the pulses could be done on the computer.

We characterized both the LuAP:Ce scintillator and the SiPM before conducting the validation experiments. The set‐up described by Ter Weele et al.^15^ was used to determine the decay profile of the scintillator. Besides the fast component with a decay time constant *τ*_d_ of approximately 17 ns, we also found a slow component with *τ*_d_ in the order of 500 ns containing ~20% of the light. Consequently, we measured the light yield with a shaping time constant of 3.0 µs following the method described by De Haas et al.^16^ Our best estimate of the light yield at 662 keV is 7.1 photons/keV, corresponding to 5.7 photons/keV in the fast component (80%). In the same experiments, we observed a very low degree of light yield nonproportionality. Therefore, values for the intrinsic resolution *R*_intr_ (and the nonproportionality factor *f*), both of which play a role in our model, were taken from work by Balcerzyk et al.,^17^ who determined them for LuAP:Ce with a low degree of nonproportionality. The values are shown in Table I.

**Table I:** Model input parameters for a LuAP:Ce scintillator (*τ*_d_ =17 ns, *Y* = 5.7 photons/keV) with good proportionality.^17^

Energy (keV)	59.5	662
Nonproportionality factor *f*	0.985	1.000
Intrinsic resolution *R*_intr_ (%)	8.5	2.8

The 1 × 1 mm^2^ SiPM had a SPAD pitch of 30 µm, so it contained 1089 SPADs, and was operated at 3.0 V and 5.0 V above the breakdown voltage of 27 V. An overview of the SiPM characteristics at these two overvoltages is shown in Table II. Note that the photodetection efficiency *η*_pd_ is a function of wavelength. The values of *η*_pd_ given in Table II were estimated on the basis of the manufacturer’s data sheet and the emission spectrum of LuAP:Ce. The values of the optical crosstalk parameter *λ* and the recharge time constant *τ*_r_ were determined from measurements of dark triggers. See supporting information for more details.

**Table II:** Model input parameters for the 1 × 1 mm^2^ SiPM (30 µm SPAD pitch) used in the validation experiments.

Overvoltage (V)	3.0	5.0
Photodetection efficiency *η*_pd_	0.410	0.455
Optical crosstalk parameter λ Recharge time constant *τ*_r_ (ns)	0.184 39.3	0.361 35.8

### 3.B Model validation: Data processing

About 2·10^5^ pulses were registered for each of the four combinations of gamma‐ray energy and SiPM overvoltage. We expect 99% of the integral under these pulses to fall within a time span of 200 ns, based on the values of *τ*_d_ and *τ*_r_. The long pulse duration is due to the large *τ*_r,_ which we purposely selected as it helps to test our model under high saturation conditions. The digitized pulses were therefore integrated using a 200 ns integration window. The pulse integrals were subsequently divided by the pulse integral of the measured single‐SPAD response (see supporting information) to obtain the equivalent number of fired SPADs *n*_f,eq_. We then generated a histogram of the resulting values and fitted a Gaussian function through the full‐energy peak to determine the mean of *n*_f,eq_ and the observed energy resolution according to Equation (12). The peaks in the histograms obtained with 662 keV gamma photons were fitted with a double Gaussian, because the full‐energy (FE) peak overlapped with the K‐escape (KE) peak (caused by 54 keV characteristic X‐rays of Lu‐atoms escaping the small crystal):

(16) $$\begin{array}{cc} y\left(n_{\text{f,eq}}\right) & =\;A_{\text{FE}}\;\exp\left(‐\frac{{\left(n_{\text{f,eq}}‐{\bar{n}}_{\text{f,eq,FE}}\right)}^{2}}{2\sigma_{\text{f,eq,FE}}^{2}}\right)\\ & \;+\;A_{\text{KE}}\;\exp\left(‐\frac{{\left(n_{\text{f,eq}}‐{\bar{n}}_{\text{f,eq,KE}}\right)}^{2}}{2\sigma_{\text{f,eq,KE}}^{2}}\right) \end{array}$$

The fit region was chosen in such a way that the following conditions were satisfied:

- Mean: ${\bar{n}}_{\text{f,eq,KE}}$ ≥ (662 − 54) / 662 · ${\bar{n}}_{\text{f,eq,FE}}$ = 0.918 · ${\bar{n}}_{\text{f,eq,FE}}$
- Energy resolution: 2.355*σ*_f,eq,KE_/${\bar{n}}_{\text{f,eq,KE}}$ ≥ 2.355*σ*_f,eq,FE_/${\bar{n}}_{\text{f,eq,FE}}$
- Amplitude: *A*_KE_ ≤ *A*_FE_

Note that the first condition contains the “≥” sign, because saturation may cause the peaks to be closer to each other than one would expect based on the energy difference between them.

We used the measurement at 3.0 V and 59.5 keV to determine the light collection efficiency *η*_lc_ of the detector – the last unknown input parameter of the model – by varying the value of *η*_lc_ in the model until the modeled mean of the equivalent number of fired SPADs coincided with the measured one. We selected this particular measurement because it is least influenced by optical crosstalk and saturation. For all other combinations of overvoltage and energy, the modeled and measured means of the equivalent number of fired SPADs and observed energy resolutions were compared to each other after running the model with this value of *η*_lc_.

Lastly, the modeled pulse shapes were experimentally validated, as these are used to derive the pulse duration *t*_95_ and the associated rate capability *r*_50_. To this end, we calculated the average shapes of the modeled and measured pulses for each combination of energy and overvoltage. The pulse amplitudes were normalized to 1 by dividing the mean pulses by their maximum value, so that they could be visually compared to each other.

### 3.C Model calculations

Once validated, the model was used to investigate the potential of SiPM‐based scintillation detectors for photon‐counting CT (PCCT). This was done by simulating the pulse shape and the associated rate capability and energy resolution for 59.5 keV X‐ray photons incident on a single, square‐shaped pixel consisting of various combinations of scintillator and SiPM. The simulations were run for light collection efficiencies *η*_lc_ of 0.50, 0.75, and 1.00 and for the following six pixel sizes: 200, 250, 333, 400, 500, and 1000 μm.

Lu_1.8_Y_0.2_SO_5_:Ce (LYSO:Ce) is a potentially suitable scintillator for PCCT because of its high density and relatively short decay time constant *τ*_d_ = 36 ns (see Table III). This scintillator is widely used in clinical positron emission tomography (PET) scanners. However, the incident photon fluence rate in CT is much higher than in PET, so an even faster scintillator could be beneficial. Therefore, LuAlO_3_:Ce (LuAP:Ce) with *τ*_d_ = 17 ns (see Table III) was also included in the study. The performance of these high‐density scintillators was first simulated in combination with sub‐mm SiPMs based on Broadcom’s NUV‐HD technology. This technology is currently available with a SPAD pitch of 30 μm. The properties of this SiPM technology are listed in Table IV. The values at an overvoltage of 3.0 V (bold face) were used for the simulations. For comparison, the values at an overvoltage of 7.0 V are also included in Table IV. The main advantage of using an overvoltage of 3.0 V is that the optical crosstalk parameter is much lower, whereas the photodetection efficiency is still decent.

**Table III:** Properties of the three fast scintillators selected for the model calculations. The required thickness is based on an X‐ray detection efficiency near 100% at 50 keV, around 90% at 100 keV and around 55% at 150 keV. The nonproportionality factors and the intrinsic resolutions apply to an X‐ray energy of 60 keV.

	LYSO:Ce^a^	LuAP:Ce^b^, ^c^	LaBr_3_:Ce^a^
Light yield *Y* (photons/keV)	33	15	63
Nonproportionality factor *f*	0.845^18^, ^19^	0.985^17^	0.980^20^, ^21^
Intrinsic resolution *R*_intr_ (%)	22^18^, ^19^	8.5^17^	8.0^20^, ^21^
Decay time constant *τ*_d_ (ns)	36	17	16
Density (g cm^‐3^)	7.1	8.3	5.1
Required thickness (mm)	1.15	1.00	3.50

^a^ Data from datasheets of Saint Gobain Crystals, unless otherwise indicated.

^b^ Data from private communication with Hilger Crystals, unless otherwise indicated.

^c^ Currently available LuAP:Ce features a slow decay component, which means that part of the 15 photons/keV is not emitted with a decay time constant of 17 ns and does not contribute to the pulse.

**Table IV:** Properties of SiPMs based on Broadcom’s NUV‐HD technology.^a^ The values in bold face were used for the model calculations. The other values are shown for comparison. Two values of *η*_pd_ are given for each combination of SPAD pitch and overvoltage: the effective photodetection efficiency for the emission spectra of the scintillators in Table III (no brackets) and the value at the wavelength of maximum sensitivity, that is, 420 nm (values in between brackets).

	SPAD pitch = 30 μm		SPAD pitch = 15 μm	
	Overvoltage 3.0 V	Overvoltage 7.0 V	Overvoltage 3.0 V	Overvoltage 7.0 V
Photodetection efficiency *η*_pd_	**0.41** (0.44)	0.48 (0.55)	0.21 (0.23)	**0.28** (0.30)
Recharge time constant *τ*_r_ (ns)	**55** ^b^	50	9.0	**7.0**
Optical crosstalk parameter *λ* ^c^	**0.1235** ^d^	0.5753	0.0128	**0.1235**

^a^ Data from Broadcom’s datasheets and from private communication with Broadcom.

^b^ The measured value of the recharge time constant in Table II differs from the values in Table IV, because the SiPM used in the experiments had tuned quenching resistors, which reduced the recharge time constant.

^c^ The datasheets mention a crosstalk probability *P*_Xtalk_, which can be converted into the crosstalk parameter *λ* using the following formula: $\lambda =\sum_{k=1}^{\infty }k\cdot {\left(P_{\text{Xtalk}}\right)}^{k}$.

^d^ The measured value of *λ* in Table II differs from the value in Table IV. This is likely due to increased optical crosstalk in the experiments caused by the presence of a scintillator covered in reflective material on top of the SiPM.^22^

In the next step, the performance of LYSO:Ce and LuAP:Ce coupled to sub‐mm SiPMs based on Broadcom’s NUV‐HD technology with a reduced SPAD pitch of 15 μm was simulated. Its properties are shown in Table IV. An overvoltage of 7.0 V (bold face) was selected in this case, because it offers the highest photodetection efficiency and the shortest recharge time constant, whereas the optical crosstalk parameter is still relatively low.

Finally, the performance of a third scintillator, LaBr_3_:Ce, coupled to sub‐mm SiPMs with a SPAD pitch of 15 μm was simulated. Table III shows that LaBr_3_:Ce is interesting due to its superior combination of decay time constant, light yield, and intrinsic energy resolution.

It was already noted that the photodetection efficiency of an SiPM is a function of wavelength. However, all three scintillators included in the present study emit in the near‐ultraviolet and blue part of the spectrum. Hence, when using the emission spectra of these three scintillators as weights to calculate the weighted average of *η*_pd_, essentially the same value is obtained each time, viz. the value shown in Table IV. For comparison, the photodetection efficiency at the wavelength of maximum sensitivity (420 nm) has been added to Table IV in between brackets.

## 4 RESULTS

In the following, we first present the results of the model validation experiments and subsequently use the validated model to compute the expected performance of SiPM‐based scintillation detectors for photon‐counting CT.

### 4.A Model validation experiments

The results of the model validation experiments are summarized in Table V (mean of the equivalent number of fired SPADs, ${\bar{n}}_{\text{f,eq}}$) and Table VI (observed energy resolution, *R*_obs,_ i.e., the energy resolution in the domain of *n*_f,eq_ as defined in Equation (12)). First, a light collection efficiency *η*_lc_ of 0.57 was determined by forcing the modeled ${\bar{n}}_{\text{f,eq}}$ to have the same value of 92.7 that was measured with 59.5 keV gamma photons at an overvoltage of 3.0 V (see Table V). The relatively low value of *η*_lc_ can have various causes, such as a suboptimally reflecting surface created by the PTFE powder and/or light losses in, or via, the optical glue used to attach the scintillator to the SiPM. A value of 29% for *R*_obs_ is expected on the basis of the model for this value of *η*_lc_, in fairly good agreement with the measured value of 33% (see Table VI). Some mismatch between model and experiment is expected, because the measured *R*_obs_ is affected by the scintillator’s intrinsic resolution *R*_intr_, in contrast to the modeled *R*_obs_. The factors contributing to *R*_intr_ have been discussed in the text below Equation (13). We may include *R*_intr_ in the modeled *R*_obs_ by backprojecting this *R*_obs_ to the domain of the number of scintillation photon‐induced triggers, followed by Pythagorean addition of *R*_intr_ (taken from Table I) and forward projection of the result to the domain of *n*_f,eq_. Both projections are based on the method described in Section 2.E. The result is a corrected value for the modeled *R*_obs_, denoted by *R*_obs_*. However, Table VI shows that this procedure does not completely bridge the gap between model and experiment.

**Table V:** Comparison of the mean equivalent number of fired SPADs ${\bar{n}}_{f,\text{eq}}$ computed using the model and measured in the validation experiments. The value of the light collection efficiency *η*_lc_ was determined by matching the modeled and measured values for an overvoltage of 3.0 V and a photon energy of 59.5 keV, that is, under conditions of low saturation and optical crosstalk.

Overvoltage (V)	3.0		5.0	
Energy (keV)	Model	Experiment	Model	Experiment
59.5	92.7	92.7	129	135
662	765	737	931	952

**Table VI:** Comparison of the observed energy resolution *R*_obs_ computed using the model and measured in the validation experiments. The measured *R*_obs_ is affected by the scintillator’s intrinsic resolution *R*_intr_, whereas the modeled *R*_obs_ is not. *R*_obs_* aims to include *R*_intr_ (from Table I) in the modeled *R*_obs_.

Overvoltage (V)	3.0			5.0		
Energy (keV)	Model *R*_obs_	Model *R*_obs_*	Experiment *R*_obs_	Model *R*_obs_	Model *R*_obs_*	Experiment *R*_obs_
59.5	29%	30%	33%	30%	31%	33%
662	6.7%	6.9%	8.0%	6.3%	6.5%	7.2%

For the other three combinations of overvoltage and energy, Table V shows that the modeled ${\bar{n}}_{\text{f,eq}}$ deviates less than 5% from the measured one. Regarding the modeled *R*_obs_* (see Table VI), a slight degradation from 30% to 31% at 59.5 keV is expected when increasing the overvoltage from 3.0 V to 5.0 V. This is because the amount of optical crosstalk increases in the model. However, this slight degradation cannot be seen in the experimental *R*_obs_. At 662 keV, a change in *R*_obs_* from 6.9% to 6.5% is expected when increasing the overvoltage, due to increasing saturation of the SiPM. A similar, but slightly more pronounced trend can be seen in the experimental *R*_obs_.

The observed differences between model and experiment may be expected in view of the assumptions in our model and the uncertainties associated with the model input parameters and the fitting of the histograms. Furthermore, we hypothesize that mismatches between modeled *R*_obs_* and measured *R*_obs_ may also be related to a non‐negligible light transport contribution (*R*_tr_ in Equation (13)) to *R*_intr_, for example due to a light leak in the detector that would also explain the relatively low value of *η*_lc_. We conclude that the model and measurements are in sufficiently good agreement to use the model for the purpose of this work, that is, to study the feasibility of using SiPM‐based scintillation detectors as a potential alternative for direct‐conversion detectors in photon‐counting CT.

**Fig. 6:**
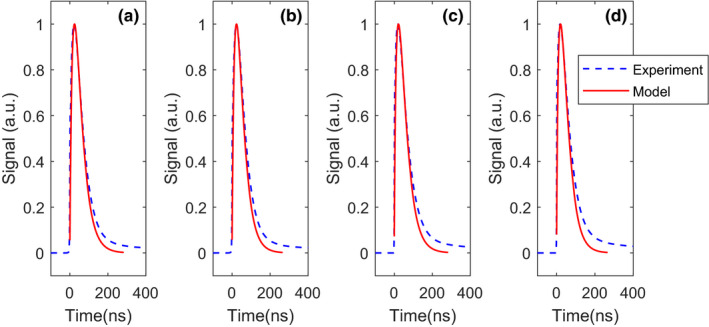
Comparison of modeled (solid red curve) and experimental (blue dashed curve) mean pulse shapes for photon energies and overvoltages of, respectively (a) 59.5 keV and 3.0 V; (b) 59.5 keV and 5.0 V; (c) 662 keV and 3.0 V; (d) 662 keV and 5.0 V. The pulse duration is relatively long, because we selected an SiPM with a large recharge time constant, which helped to test our model under high saturation conditions.

Fig. 6 shows the mean pulse shapes derived from the four validation experiments in comparison to the mean pulse shapes according to the corresponding model calculations. Regardless of overvoltage and energy, the simulated pulse shapes nicely follow the experimental ones in the main part of the pulse. Deviations can be observed in the tails, where the experimental mean pulse shapes return to the baseline more slowly. This is due to the slow decay component that we observed for the LuAP:Ce scintillator used in the experiments (see Section 3.A), which is not accounted for by the model. Slow components are not uncommon in scintillators and can often be reduced considerably through proper engineering of the scintillation material. It is evident that the minimization of slow components is important if a scintillator is to be used in a high count‐rate application like photon‐counting CT. In case slow scintillation decay components cannot be fully eliminated, implementing a baseline restorer in the read‐out circuit is a possible remedy.

### 4.B Model calculations

**Fig. 7:**
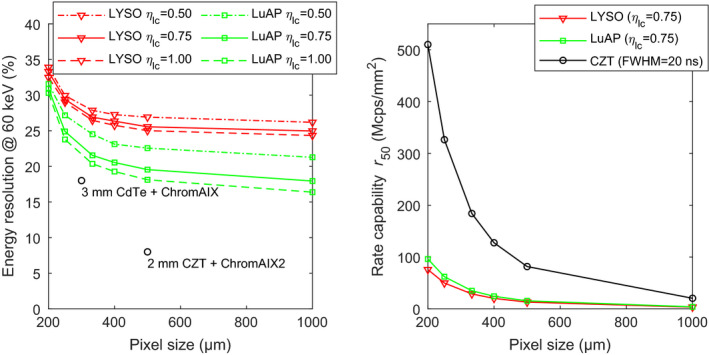
Energy resolution at 60 keV (left) and rate capability *r*_50_ (right), both as a function of pixel size, for LYSO:Ce and LuAP:Ce scintillators coupled to the SiPMs with a SPAD pitch of 30 μm. For comparison, data reported for CdTe‐ and CZT‐based photon‐counting detectors are also shown.^23^, ^24^

Fig. 7 shows the expected performance of sub‐mm pixels of LYSO:Ce and LuAP:Ce scintillators coupled to the SiPMs with a SPAD pitch of 30 μm. It can be observed that LuAP:Ce outperforms LYSO:Ce, both in terms of rate capability and energy resolution. The former was expected, because of the shorter decay time constant. The latter shows the importance of using scintillators with a low degree of nonproportionality and associated good intrinsic resolution *R*_intr_. As shown in Table III, the light yield of LuAP:Ce is at least two times lower than that of LYSO:Ce. However, its *R*_intr_ is much better, which compensates for the worse *R*_stat_ that results from its lower light yield (see Equation (13)). Fig. 7 (left) furthermore illustrates the importance of a high light collection efficiency *η*_lc_ for achieving good energy resolution. Finally, it can be observed that both scintillators suffer from a degradation of the energy resolution with decreasing pixel size. This trend is due to increasing saturation of the SiPM.

To put these results into perspective, Fig. 7 (left) shows two data points reported for CdTe‐ and CZT‐based X‐ray photon‐counting detectors from Philips.^23^, ^24^ Clearly, it is impossible to get near these data points with an LYSO:Ce‐based detector. However, we observe that a LuAP:Ce‐based detector with high *η*_lc_ could get close to the “3 mm CdTe + ChromAIX” data point if the saturation effect were absent.

The plots of the rate capability *r*_50_ in Fig. 7 (right) can be compared to the same Philips detectors. These detectors output Gaussian shaped pulses, so 95% of the total area under the pulses falls within four standard deviations. Given that the pulses have an FWHM of 20 ns,^24^ their *t*_95_ equals 34 ns. The *r*_50_ curve for CZT in Fig. 7 (right) is based on this number. Clearly, the rate capability of the combinations of scintillator and SiPM shown in Fig. 7 (right) is far from the rate capability of the CZT detector. Note that only the curve for *η*_lc_ = 0.75 is shown, as the value of *η*_lc_ hardly affects the pulse duration and the associated rate capability *r*_50_.

It can be concluded from the above results that less saturation and shorter pulses are required to be competitive with room‐temperature semiconductor detectors. Detectors based on the advanced SiPMs with a SPAD pitch of 15 μm could potentially achieve this, because these SiPMs have a much shorter recharge time constant and a quadrupled number of SPADs for a given pixel size.

**Fig. 8:**
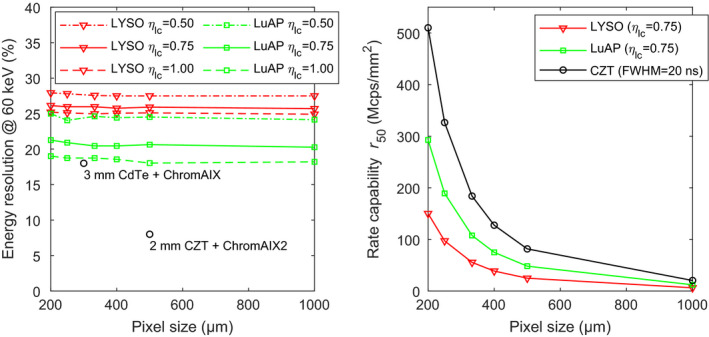
Energy resolution at 60 keV (left) and rate capability *r*_50_ (right), both as a function of pixel size, for LYSO:Ce and LuAP:Ce scintillators coupled to the advanced SiPMs with a SPAD pitch of 15 μm. For comparison, data reported for CdTe‐ and CZT‐based photon‐counting detectors are also shown.^23^, ^24^

The data in Fig. 8 (left) indeed show that sub‐mm pixels of these scintillators coupled to the SiPMs with a SPAD pitch of 15 μm hardly suffer from degradation of the energy resolution with decreasing pixel size. However, for the pixel sizes that are least affected by saturation (e.g., 1000 µm), a comparison of Fig. 7 (left) and Fig. 8 (left) shows that the use of SiPMs with a SPAD pitch of 15 µm results in a slight worsening of the energy resolution, due to the lower photodetection efficiency of these SiPMs (see Table IV). This effect is less pronounced for LYSO:Ce, because the energy resolution obtained with this material is mainly determined by its relatively poor intrinsic resolution *R*_intr_.

It can be appreciated from Fig. 8 (right) that the shorter recharge time constant of the advanced SiPMs with a SPAD pitch of 15 µm leads to a considerable improvement of the rate capability. The value of the mean pulse duration *t*_95_ improves from 180 ns to slightly less than 60 ns for LuAP:Ce, which is in the same order of magnitude as the *t*_95_ of the CZT detector (34 ns). For pixel sizes ranging from 200 µm to 500 µm, a LuAP:Ce pixel would have to be made approximately 100 µm smaller than a CZT pixel of a given size to achieve the same rate capability. For example, a 400 µm LuAP:Ce pixel would have a similar rate capability as a 500 µm CZT pixel.

**Fig. 9:**
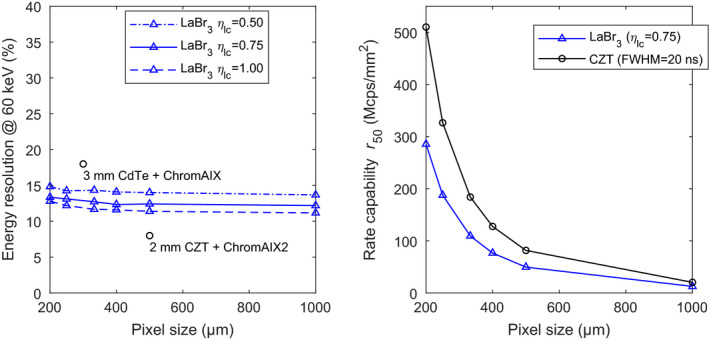
Energy resolution at 60 keV (left) and rate capability *r*_50_ (right), both as a function of pixel size, for LaBr_3_:Ce scintillators coupled to the advanced SiPMs with a SPAD pitch of 15 μm. For comparison, data reported for CdTe‐ and CZT‐based photon‐counting detectors are also shown.^23^, ^24^

The modeled performance of detectors consisting of sub‐mm LaBr_3_:Ce crystals coupled to the advanced SiPMs with a SPAD pitch of 15 μm is graphically displayed in Fig. 9. The decay time constant of LaBr_3_:Ce is 16 ns, which is only 1 ns shorter than the one of LuAP:Ce. As a result, the rate capability curve in Fig. 9 (right) is very similar to the one of LuAP:Ce in Fig. 8 (right). Thus, a 400 µm LaBr_3_:Ce pixel also has a similar rate capability as a 500 µm CZT pixel, for example. However, a comparison of Fig. 8 (left) and Fig. 9 (left) shows that LaBr_3_:Ce substantially outperforms LuAP:Ce in terms of energy resolution. The achievable values of the energy resolution at 60 keV lie in between the two data points reported for the CdTe and CZT detectors. Furthermore, only a slight degradation of the energy resolution with decreasing pixel size is observed, which indicates that sub‐mm pixels of the SiPMs with a SPAD pitch of 15 μm hardly suffer from saturation, even when coupled to a scintillator with a very high light yield such as LaBr_3_:Ce.

**Fig. 10:**
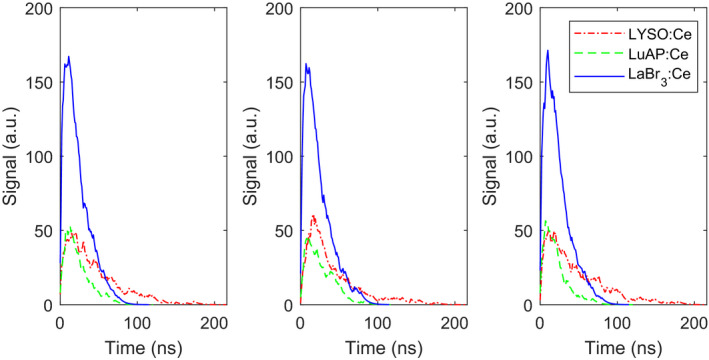
Examples of simulated pulse shapes generated by 333 μm pixels of LaBr_3_:Ce (solid blue curve), LuAP:Ce (dashed green curve), and LYSO:Ce (dash‐dotted red curve) coupled to the advanced SiPMs with a SPAD pitch of 15 μm, in response to 59.5 keV X‐ray photons and for a light collection efficiency of 0.75.

Lastly, Fig. 10 shows several examples of pulses generated by 333 μm pixels of the three scintillators when coupled to the advanced SiPMs with a SPAD pitch of 15 µm, in response to 59.5 keV photons and assuming a light collection efficiency of 0.75. LaBr_3_:Ce has a much higher light yield than LuAP:Ce, but a very similar decay time constant. This leads to a much higher pulse amplitude, but 95% of the area under the curve falls within the same window of 57‐58 ns (*t*_95_) for both scintillators. LYSO:Ce also has a higher light yield than LuAP:Ce, but its decay time constant is considerably larger. Thus, the scintillation photons from LYSO:Ce are more spread out in time, which results in a similar pulse amplitude, but a much longer *t*_95_ of 113 ns.

## 5 DISCUSSION

SiPMs exhibit a nonproportional response, mainly due to the occurrence of saturation and optical crosstalk. This may substantially affect the performance of SiPM‐based scintillation detectors with sub‐mm pixel sizes for photon‐counting CT. We therefore incorporated detailed descriptions of saturation and optical crosstalk in our model and compared the resulting model calculation to a series of validation experiments, some of which were performed under conditions in which these phenomena have a substantial influence on the measured result. Since the smallest SiPM size currently available is 1 × 1 mm^2^, high saturation conditions were effectuated by irradiating a 0.9 × 0.9 × 1.0 mm^3^ LuAP:Ce crystal coupled to such a 1 × 1 mm^2^ SiPM with 662 keV gamma photons, in addition to irradiations with clinically more relevant 60 keV gamma photons. Good agreement between model and experiment was found in both cases, especially when the multitude of model input parameters and assumptions is considered. This gives confidence about the predictive power of the model.

According to our model calculations, LuAP:Ce and LaBr_3_:Ce appear to be promising alternatives for CdTe‐ and CZT‐based detectors when coupled to SiPMs with a small SPAD pitch and a very short recharge time constant, such as the advanced SiPM prototype considered in this work. While LuAP:Ce and LaBr_3_:Ce have similarly high count rate capabilities, LaBr_3_:Ce offers the best energy resolution. It should be noted that LaBr_3_:Ce is hygroscopic and needs special treatment and packaging before it can be used outside a moisture‐free environment. It is nevertheless used in a wide variety of detectors and applications, as is the case for other hygroscopic crystals such as NaI:Tl. We thus see LaBr_3_:Ce as the most promising of the investigated materials for photon‐counting CT applications in which the best possible count rate capability and energy resolution are required. Nevertheless, LuAP:Ce and LYSO:Ce could be useful alternatives in less demanding X‐ray photon‐counting applications.

Our model calculations furthermore indicate that rate capabilities adequate for photon‐counting CT (PCCT) can be achieved when the detector pixel size equals 400 μm or less. Such pixel sizes also fulfill the spatial resolution requirements of PCCT. Yet, two challenges arise when developing a scintillation detector with such small pixels, which will need to be addressed in future work.

The first challenge concerns the escape of fluorescence and/or Compton‐scattered X‐rays from a detector pixel and their possible absorption in neighboring pixels (X‐ray crosstalk). One of the detector properties that affect this phenomenon is the atomic number of the elements present in the detection material. Table VII shows the characteristics of the K‐shell X‐ray fluorescence of a few relevant elements, for example. However, detailed modelling of the effect of secondary X‐rays on the detector performance requires Monte Carlo particle tracking software. Combining the distribution of energy depositions in a pixel obtained from Monte Carlo simulations with the detector response model presented in this work will yield the full spectral response of a detector pixel, for example.

**Table VII:** Characteristics of the K‐shell X‐ray fluorescence of a few relevant elements. If the incident X‐ray photon energy exceeds the K‐edge energy, K‐shell photo‐electric absorption may take place. The K fluorescence yield describes which fraction of these absorptions leads to the emission of a K X‐ray with energy in the tabulated range. The numbers are based on data from the NIST standard reference database 128.

Detection material	LaBr_3_	LuAP	LYSO	CdTe	
Element	La	Lu	Lu	Cd	Te
K‐edge energy (keV)	38.9	63.3	63.3	26.7	31.8
K fluorescence yield	0.91	0.96	0.96	0.84	0.88
Energy range of K_α1_, K_α2_, K_β1_ X‐rays (keV)	33.0–37.8	53.0–61.3	53.0–61.3	23.0–26.1	27.2–31.0

Second, scintillator‐based PCCT detectors need a form of optical isolation in between the pixels in order to avoid light sharing between neighboring pixels (Fig. 1(a)). The use of conventional reflectors with a thickness of ~100 μm can lead to a relatively large dead area and loss of dose efficiency in the case of sub‐mm pixels. On the other hand, such reflectors often provide excellent optical isolation. Distortion of the measurement of counts and energy, as caused by charge sharing in direct‐conversion detectors, can thus be avoided. Moreover, thinner reflectors of excellent quality exist nowadays. In particular, reflectors ranging from 38 µm to 65 µm in thickness have been used in high resolution imaging detectors based on scintillation crystals ranging from 220 µm to 430 µm in size.^25^, ^26^, ^27^ Furthermore, innovative optical isolation techniques are under development. For example, LaBr_3_:Ce has been grown with a columnar microstructure,^28^ like the CsI:Tl scintillator used in flat panel detectors for digital radiography. In research laboratories, laser‐induced optical barriers have been created in a variety of scintillators, including LYSO:Ce.^29^, ^30^ Both techniques come with essentially no dead area, but the optical isolation may be less than what is achievable with physical reflectors. It thus needs to be evaluated if the positive effect of having zero dead area outweighs the negative effect of having some light sharing (cf. charge sharing in direct‐conversion detectors).

**Fig. 11:**
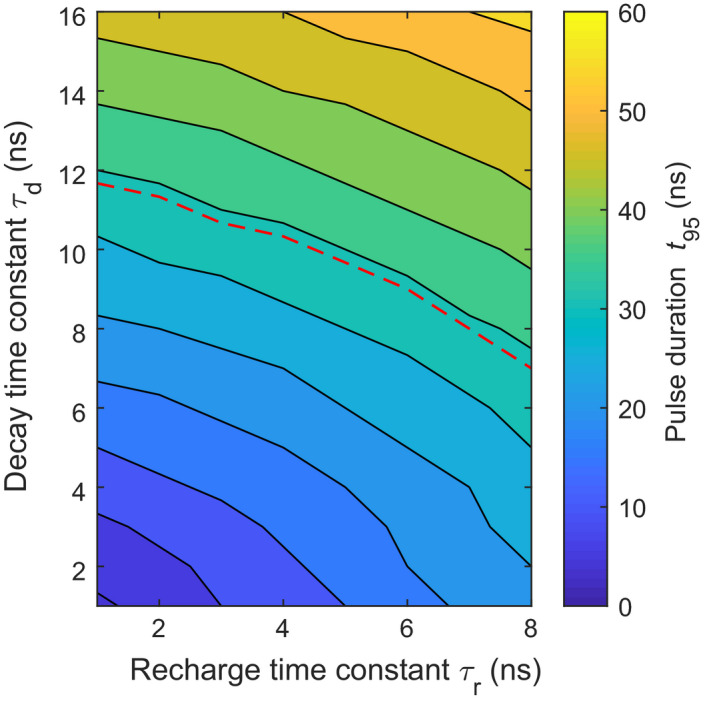
Expected pulse duration *t*_95_ as a function of the decay time constant *τ*_d_ of the scintillator and the recharge time constant *τ*_r_ of the SiPM. The red‐dashed curve indicates the combinations of *τ*_d_ and *τ*_r_ that yield *t*_95_ = 34 ns, which is the pulse duration of the state‐of‐the‐art CdTe and CZT detectors to which we compared our results in Section 4.B.^23^, ^24^

Lastly, we note that there may be potential to further improve the rate capability of SiPM‐based scintillation detectors. The pulse duration *t*_95_ is mainly determined by the decay time constant *τ*_d_ of the scintillator and the recharge time constant *τ*_r_ of the SiPM. The best values of these parameters encountered in the present work are 16 ns and 7 ns, respectively. However, ongoing research into fast scintillators and SiPMs may yield even smaller time constants. Fig. 11 shows the expected *t*_95_ calculated using our model, as a function of *τ*_d_ and *τ*_r_. For combinations of *τ*_d_ and *τ*_r_ that end up below the red‐dashed curve, *t*_95_ ≤ 34 ns. In other words, such detectors output shorter pulses than the state‐of‐the‐art CdTe and CZT detectors to which we compared our results in Section 4.B.^23^, ^24^

## 6 CONCLUSION

In this work, we investigate the feasibility of developing SiPM‐based scintillation detectors for use in photon‐counting CT (PCCT) scanners. To this end, we introduce and experimentally validate a model that can be used to compute the expected energy resolution as well as the expected pulse shape and associated rate capability of such detectors. The model accounts for SiPM saturation and optical crosstalk, as these phenomena may substantially affect the performance of detector pixels with sub‐mm dimensions. Such small pixels are required to (1) handle the high incident X‐ray photon fluence rate and (2) provide sufficient spatial resolution for PCCT.

Our model calculations indicate that the energy resolution of sub‐mm pixels of fast and bright scintillators, such as LYSO:Ce, LuAP:Ce, and LaBr_3_:Ce, when coupled to currently available SiPMs with a SPAD pitch of 30 μm, degrades with decreasing pixel size as a result of SiPM saturation. Moreover, the recharge time constant of about 55 ns of these SiPMs has a dominant influence on the detector pulse shape, resulting in relatively long detector pulse durations of about 200 ns.

Scintillation detectors based on more advanced SiPMs, having a SPAD pitch of 15 μm and a recharge time constant of only 7 ns, appear to have much more favorable properties. In particular, LuAP:Ce and LaBr_3_:Ce detectors utilizing such SiPMs will generate output pulses that last slightly less than 60 ns, which is in the same order of magnitude as the pulse duration of current PCCT direct‐conversion detectors. In particular, scintillation detectors with a pixel size of about 400 μm or less can yield rate capabilities comparable to typical CdTe and CZT detectors with a pixel size of about 500 μm. Moreover, an SiPM‐based LaBr_3_:Ce detector can achieve an energy resolution of 11.5%‐13.5% at 60 keV. These numbers also compare well with those of CdTe and CZT detectors.

Based on the current findings, we conclude that it may be feasible to develop SiPM‐based scintillation detectors for photon‐counting CT that can compete with CdTe and CZT detectors in terms of energy resolution and rate capability.

## CONFLICT OF INTEREST

The authors have no relevant conflict of interest to disclose.

## Supporting information


**Figure S1**. Illustration of the method used to determine the optical crosstalk parameter *λ*. (a) A histogram of measured dark pulse integrals shows several equally‐spaced peaks indicated by the arrows. The fraction of events in each peak is determined using the equally spaced vertical red lines as borders between the peaks. (b) The red curve is a fit of the Borel distribution with *n*_tr.oc_=*k* and fitting parameter *λ* (equation (3) of the main text) through the measured fraction of events as a function of the peak number *k*. The value of *λ* was determined from this fit.

**Figure S2**. The mean pulse shape of the single‐SPAD response on (a) linear scale and (b) logarithmic scale. An exponentially decaying function with the recharge time constant *τ*_r_ as a fitting parameter was fitted through the tail of the pulse in order to determine the value of *τ*_r_.

Supplementary Material

## Data Availability

The data that support the findings of this study are available from the corresponding author upon reasonable request.
