# Supplementary figures and images for: Silicon photomultiplier‐based scintillation detectors for photon‐counting CT: A feasibility study
Source: Med Phys. 2021 Jun 25;48(10):6324–38. doi: 10.1002/mp.14886 (PMC8596580; doi:10.1002/mp.14886)

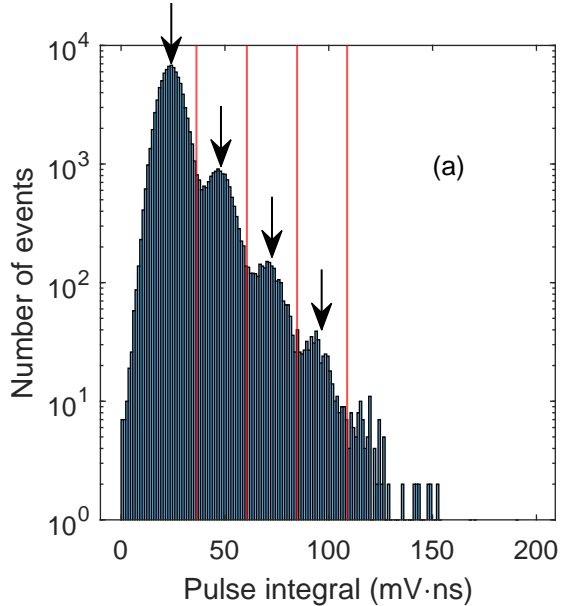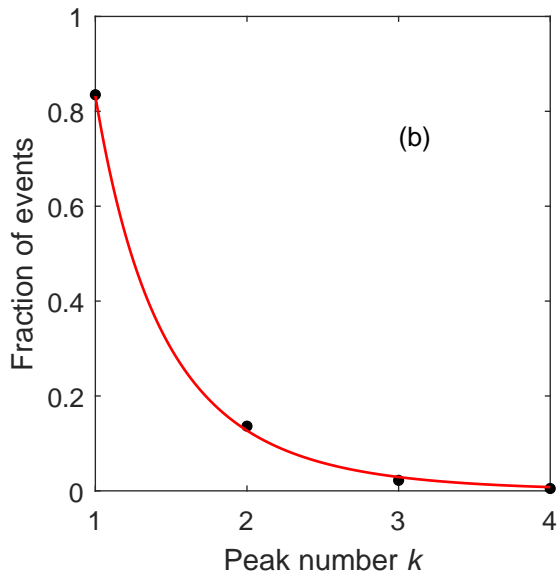

Supplement: Supplementary file 1 — Figure S1. Illustration of the method used to determine the optical crosstalk parameter λ. (a) A histogram of measured dark pulse integrals shows several equally‐spaced peaks indicated by the arrows. The fraction of events in each peak is determined using the equally spaced vertical red lines as borders between the peaks. (b) The red curve is a fit of the Borel distribution with n tr.oc=k and fitting parameter λ (equation (3) of the main text) through the measured fraction of events as a function of the peak number k. The value of λ was determined from this fit. [file MP-48-6324-s001.pdf]

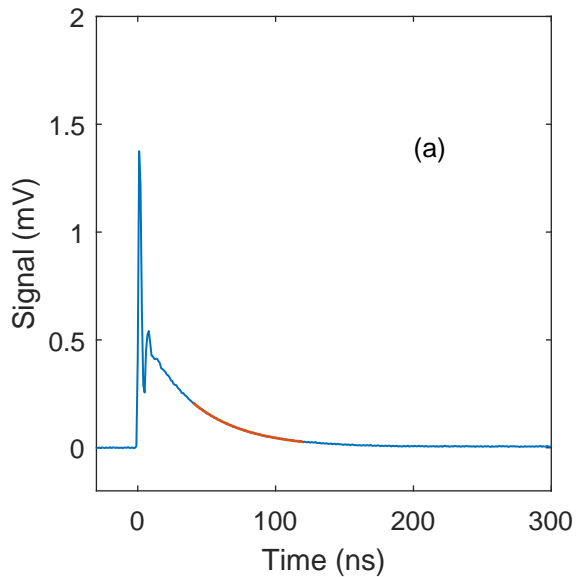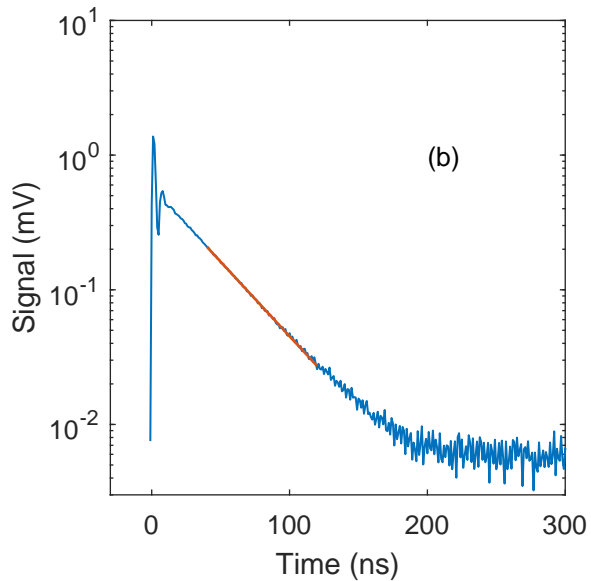

Supplement: Supplementary file 2 — Figure S2. The mean pulse shape of the single‐SPAD response on (a) linear scale and (b) logarithmic scale. An exponentially decaying function with the recharge time constant τ r as a fitting parameter was fitted through the tail of the pulse in order to determine the value of τ r. [file MP-48-6324-s002.pdf]
