# Supplementary material for: Silicon photomultiplier‐based scintillation detectors for photon‐counting CT: A feasibility study
Source: Med Phys. 2021 Jun 25;48(10):6324–38. doi: 10.1002/mp.14886 (PMC8596580; doi:10.1002/mp.14886)
Supplement: Supplementary file 3 — Supplementary Material [file MP-48-6324-s003.doc]

**Supporting information**

This supporting information discusses how the optical crosstalk parameter λ, the mean single-SPAD response, and the recharge time constant *τ*_r_ of the 1×1 mm^2^ SiPM that was used in the model validation experiments were determined from a measurement of dark triggers.

The detector was placed in a light-tight box and not exposed to ionizing radiation for the measurement of dark triggers. The 0.9×0.9×1.0 mm^3^ LuAP:Ce scintillation crystal was glued to the SiPM and covered in PTFE powder, because these conditions are expected to increase the number of crosstalk photon-induced triggers per dark trigger compared to a bare SiPM.^1^ The SiPM was operated at an overvoltage of 3.0 V. A histogram of the integrals of about 10^5^ recorded dark pulses was generated. As shown in figure S1, such a histogram shows several equally spaced peaks. The *k*^th^ peak corresponds to a dark trigger plus *k*-1 crosstalk photon-induced triggers. The Borel distribution with *n*_tr.oc_=*k* and fitting parameter *λ* (see equation (3) of the main text) was fitted through the fraction of events in each peak in order to determine the value of *λ*. A value of 0.184 was found.

The pulses of the events in the central part of the first peak were used to determine the mean pulse shape of the single-SPAD-response. As shown in figure S2, an exponentially decaying function with the recharge time constant *τ*_r_ as a fitting parameter was fitted through the tail of that pulse in order to determine the value of *τ*_r_. A value of 39.3 ns was found.

**Figure captions**

Figure S1. Illustration of the method used to determine the optical crosstalk parameter *λ*. (a) A histogram of measured dark pulse integrals shows several equally-spaced peaks indicated by the arrows. The fraction of events in each peak is determined using the equally spaced vertical red lines as borders between the peaks. (b) The red curve is a fit of the Borel distribution with *n*_tr.oc_=*k* and fitting parameter *λ* (equation (3) of the main text) through the measured fraction of events as a function of the peak number *k*. The value of *λ* was determined from this fit.

Figure S2. The mean pulse shape of the single-SPAD response on (a) linear scale and (b) logarithmic scale. An exponentially decaying function with the recharge time constant *τ*_r_ as a fitting parameter was fitted through the tail of the pulse in order to determine the value of *τ*_r_.

**References**

1. Gola A, Ferri A, Tarolli A, Zorzi N, Piemonte C. SiPM optical crosstalk amplification due to scintillator crystal: effects on timing performance*. Physics in Medicine & Biolo*gy. 2014;59(13):3615-3635. doi:10.1088/0031-9155/59/13/3615
